# Comparative transcriptomics uncovers alternative splicing changes and signatures of selection from maize improvement

**DOI:** 10.1186/s12864-015-1582-5

**Published:** 2015-05-08

**Authors:** Jun Huang, Youjun Gao, Haitao Jia, Lei Liu, Dan Zhang, Zuxin Zhang

**Affiliations:** National Key Laboratory of Crop Genetic Improvement, Huazhong Agricultural University, Wuhan, 430070 China; Hubei Collaborative Innovation Center for Grain Crops, Jingzhou, 434025 China

**Keywords:** Transcriptome, Alternative splicing, *Zea mays* ssp. *parviglumis*, *Zea mays* ssp. *mexicana*, Biotic and abiotic stresses

## Abstract

**Background:**

Alternative splicing (AS) is an important regulatory mechanism that greatly contributes to eukaryotic transcriptome diversity. A substantial amount of evidence has demonstrated that AS complexity is relevant to eukaryotic evolution, development, adaptation, and complexity. In this study, six teosinte and ten maize transcriptomes were sequenced to analyze AS changes and signatures of selection in maize domestication and improvement.

**Results:**

In maize and teosinte, 13,593 highly conserved genes, including 12,030 multiexonic genes, were detected. By identifying AS isoforms from mutliexonic genes, we found that AS types were not significantly different between maize and teosinte. In addition, the two main AS types (intron retention and alternative acceptor) contributed to more than 60% of the AS events in the two species, but the average unique AS events per each alternatively spliced gene in maize (4.12) was higher than that in teosinte (2.26). Moreover, 94 genes generating 98 retained introns with transposable element (TE) sequences were detected in maize, which is far more than 9 retained introns with TEs detected in teosinte. This indicates that TE insertion might be an important mechanism for intron retention in maize. Additionally, the AS levels of 3864 genes were significantly different between maize and teosinte. Of these, 151 AS level-altered genes that are involved in transcriptional regulation and in stress responses are located in regions that have been targets of selection during maize improvement. These genes were inferred to be putatively improved genes.

**Conclusions:**

We suggest that both maize and teosinte share similar AS mechanisms, but more genes have increased AS complexity during domestication from teosinte to maize. Importantly, a subset of AS level-increased genes that encode transcription factors and stress-responsive proteins may have been selected during maize improvement.

**Electronic supplementary material:**

The online version of this article (doi:10.1186/s12864-015-1582-5) contains supplementary material, which is available to authorized users.

## Background

Precursor mRNA (pre-mRNA) splicing is an important step in eukaryotic gene expression that plays a crucial role in higher eukaryotic developmental regulation and environmental stress response [1]. After precursor mRNA splicing, introns are removed and exons are ligated into one or more mature transcripts or isoforms. Since Walter Gilbert [2] first postulated that alternative splicing (AS) can create different isoforms from a common template, increasing evidence has shown that AS widely occurs in animals and plants. For example, transcripts from 92%–94% of human intron-containing genes and approximately 61% of *Arabidopsis* multiexonic genes can be alternatively spliced [3,4]. In addition, approximately 63% and 51% of intron-containing genes also undergo AS in soybean [5] and maize [6], respectively.

In general, AS events can occur via four different mechanisms: exon skipping (ES), alternative donor site (AD), alternative acceptor site (AA), and intron retention (IR). However, there are also complex AS types combined with at least two simple forms or some other low frequent forms such as mutually exclusive exons, alternative transcription start sites, and multiple polyadenylation sites in eukaryotes [7]. However, the frequency of each AS event is different and can be gene- and species-dependent. In animals, ES is the most frequent AS event and IR is the least frequent; for example, approximately 35.2% of all AS events in humans are caused by by ES, but only 0.01% are caused by IR [3]. In contrast, in *Arabidopsis* [4], maize [6], *Brachypodium*, [8] and cotton [9], IR is the predominant form of AS, whereas ES only accounts for a small proportion of AS events.

AS is involved in a wide array of biological functions, particularly in response to biotic and abiotic stress. In humans, approximately 15% of inherited diseases are caused by mutations that interfere with mRNA splicing [10]. In *Arabidopsis*, AS is strongly associated with environmental stimuli [11]. For example, isoforms retaining the fourth intron of *CCA1* increased under high light conditions but decreased under low temperature conditions [12]. Recent studies found that diverse mRNA isoforms produced from a given gene can accelerate genome evolution by generating new functions. A splice site mutation in the fourth intron of sorghum *Shattering1* (*Sh1*) results in the removal of the fourth exon, and this variation underwent selection during the sorghum domestication and improvement [13].

Maize (*Zea mays* ssp. *mays*), which is a model plant for studying crop domestication, evolved from the annual teosinte *Zea mays* ssp. *parviglumis* approximately 6,000 to 10,000 years ago and greatly differs in morphology from its progenitors [14,15]. Recently, several key domestication-related genes that are responsible for the morphological changes between maize and teosinte were identified, including *tga1* [16], *tb1* [17], *ba1* [18], *ra1* [19], and *zfl2* [20]. DNA sequencing advances have led to the discovery of thousands of genes with strong signals of selection [21,22]. At the transcriptional level, Swanson-Wagner et al. [23] found hundreds of genes that have altered expression levels or co-expression profiles during domestication. Some of the genes involved in responding to biotic and abiotic stresses were significantly enriched with selective sweeps. In addition, Lemmon et al. [24] suggested that gene expression may be caused by the modification of *cis* regulatory elements. They found that approximately 4% of genes showing evidence for consistent *cis* regulatory divergence that differentiates maize from teosinte were significantly correlated with maize domestication and improvement.

Early studies on maize domestication were primarily focused on nucleotide sequence diversity and expression changes between maize and teosinte, but the differences on alternatively spliced genes (AS genes), alternative splicing types (AS types), AS events and level between maize and teosinte, and evolutionary role of these differences has been poorly studied. To better understand the evolutionary role of AS in maize domestication, the transcriptome of the seedling stage from six teosinte accessions and ten maize inbred lines were sequenced. Furthermore, genome-wide AS profiles were analyzed, and AS genes, types, events, and levels were compared between maize and teosinte. We found that maize had greater AS complexity than teosinte, and a subset of AS level-altered genes were enriched in transcriptional regulation and stress responses. This subset was located in regions that have been targets of selection for maize improvement.

## Results

### Transcriptome sequencing and assembly

To assemble the transcriptome of maize and teosinte, cDNA libraries of ten maize inbred lines, three *Zea mays* ssp*. parviglumis* and three *Zea mays* ssp*. mexicana* accessions (Additional file 1: Table S1) were sequenced with the Solexa sequencing platform. In total, 728.7 million reads (53.8 Gb) were obtained, with an average of 45.5 million reads (~3.3 Gb) per sample. After discarding low quality reads, 607.2 million high quality reads (~83.3% of total reads) were used for further analysis. Without a reference genome, high quality reads of teosinte were first assembled *de novo*. After removing redundant sequences, 55,069 to 95,668 contigs were reconstructed in six teosinte libraries. The length of contigs varied from 100 to 8,770 bp, the N50 and N90 of teosinte transcriptomes ranged from 493 to 937 bp and 151 to 217 bp, respectively (Additional file 1: Table S2). For maize inbred lines, transcriptomes were assembled based on B73 reference genome: 39,441 to 53,105 isoforms were assembled and the N50 and N90 of maize transcriptomes ranged from 1,314 to 1,890 bp and 467 to 843 bp, respectively (Additional file 1: Table S3). For a global view of transcriptomes of the two teosinte subspecies, we assembled pseudo-transcriptomes by clustering contigs from three *Zea mays* ssp. *parviglumis* and three *Zea mays* ssp*. mexicana* libraries, respectively. Totals of 118,886 and 123,759 unique isoforms for *Zea mays* ssp. *parviglumis* and *Zea mays* ssp. *mexicana,* respectively, were obtained; the longest transcript in *Zea mays* ssp*. parviglumis* and in *Zea mays* ssp. *mexicana* was 8,901 and 8,296 bp, respectively. Under a 95% identity and coverage cutoff, *Zea mays* ssp*. parviglumis* and *Zea mays* ssp. *mexicana* transcripts could be perfectly matched with 22,406 and 22,214 maize reference genes, respectively, and 19,378 genes were shared by the two teosinte subspecies. High homology of isoforms between maize and teosinte indicated high conservation of proteins encoded by function genes during maize domestication. This finding is consistent with the study on expression and *cis*-regulatory changes between maize and teosinte [23,24].

### Variant and genetic diversity characterization

**Figure 1:**
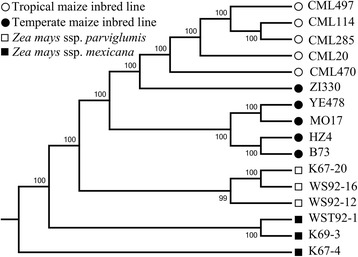
Phylogenetic relationships among 16 sequenced samples. Taxa in the neighbor-joining tree are represented by different shapes. Empty circles denote tropical maize inbred lines; solid circles denote temperate maize inbred lines; empty squares denote accessions of *Zea mays* ssp. *parviglumis*; and solid squares denote accessions of *Zea mays* ssp. *mexicana*. The phylogenetic analysis was performed using the neighbor-joining method with 1,000 bootstrap replicates in the PHYLIP package.

To characterize genetic diversity among all sequenced samples, clean reads from each sample were mapped to the B73 reference genome, respectively. After discarding the low coverage and quality SNPs and Indels, we detected 588,971 SNPs from 26,586 genes with 1–242 variant sites in a single gene (Additional file 2: Figure S1A), and 270,024 Indels with a −66 to 65 bp deletion or insertion that was distributed from −20 to 20 bp (Additional file 2: Figure S1B). Of the SNPs, 376,620 were transitions and 212,351 were transversions. The ratio of transitions to transversions was approximately 1.77 and likely caused by the sequencing coverage and filtering steps. Moreover, 371,651 SNPs were located in exons, 149,307 were in introns, and 97,900 were in intergenic regions. These results indicate that some transcribed sequences may not be annotated in the B73 filtered gene set or some unknown fragments were transcribed in our transcriptome data. Phylogenetic analysis using all SNPs detected in this study showed that maize inbred lines, *Zea mays* ssp. *parviglumis*, and *Zea mays* ssp*. mexicana* accessions clearly grouped into three clusters. Temperate and tropical maize lines also clearly grouped into different clades (Figure 1). This result is consistent with previous studies that have used DNA markers [14].

### Orthologous gene identification

**Figure 2:**
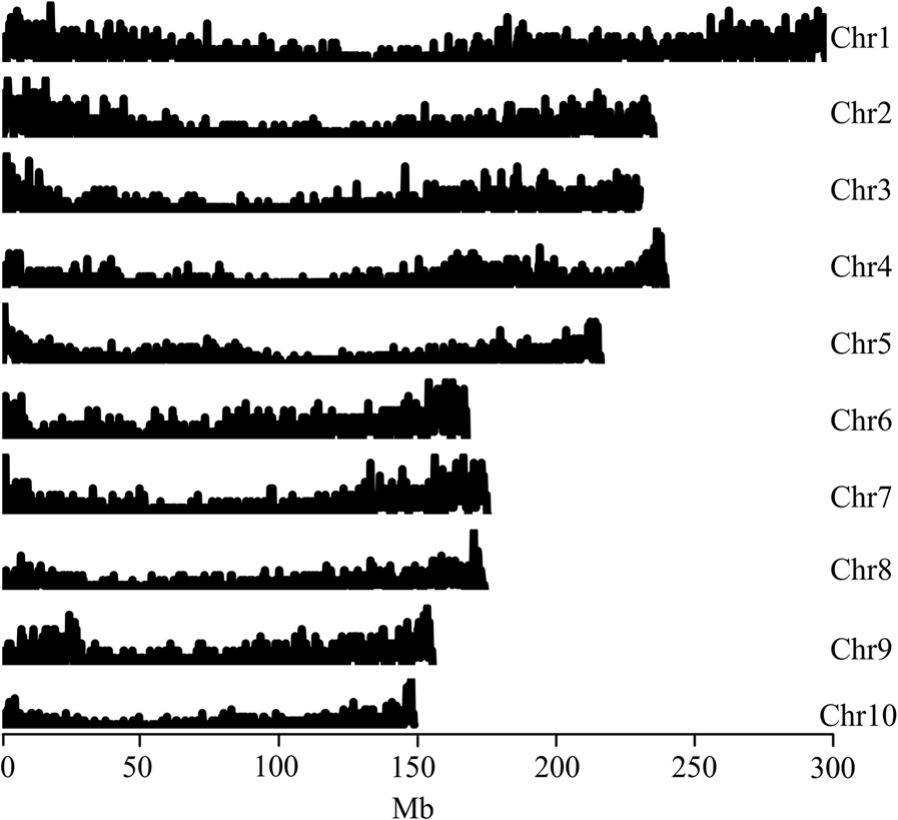
Orthologous gene distribution in the maize genome. Gene density statistics were calculated with a 200-kb sliding-window. The X-axis denotes the physical position of a chromosome. The Y-axis denotes the number of genes located in this sliding window. Chr = chromosome.

*Zea mays* ssp. *parviglumis* and *Zea mays* ssp. *mexicana* pseudo-transcriptomes were used to identify orthologous gene pairs between maize and teosinte using the reciprocal best BLAST hit method. A Total of 16,594 and 17,052 high confidence orthologs of maize with an e-value ≤ 1E-10 were identified in *Zea mays* ssp*. parviglumis* and *Zea mays* ssp. *mexicana*, respectively. Of these, 13,593 orthologs that accounted for 34.27% of the B73 filtered gene set were shared between the two species and distributed across all 10 maize chromosomes. Overall, ortholog density was low in centromere-proximal regions and high in chromosome arms (Figure 2). These shared orthologs were highly conserved during maize domestication; therefore, these shared orthologs were further used for AS landscape of maize and teosinte transcriptome analysis.

### Maize and teosinte alternative splicing landscapes

**Figure 3:**
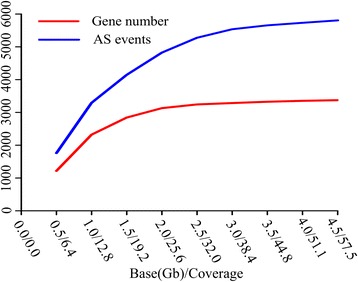
Relationship between sequencing depth and alternative splicing. The X-axis denotes the sequencing depth and average gene coverage. The Y-axis denotes the numbe of alternatively spliced genes or number of alternative splicing events.

To determine the relationship between sequencing depth and AS detection power, the sequencing library (Ye478) was first used to randomly create sub-libraries to detect AS transcripts. Overall, sequencing depth was highly positively correlated with the average coverage of each gene, and positively correlated with the AS gene number and AS events, in particular, when sequencing depth is less than 2.5 Gb (Figure 3). However, when sequencing depth was more than 2.5 Gb, the increase of sequencing depth did not significantly increase the number of AS genes and all AS events (Figure 3). This result is consistent with Liu’s findings in *Arabidopsis* [25]. In this study, sequencing depth of most libraries was more than 2.5 Gb, and implicating our sequencing data was well to support the identification of AS transcripts.

To characterize the maize and teosinte AS landscapes, AS events that occurred in all shared orthologs of each sample were evaluated. In 13,593 shared genes, 12,030 genes were annotated with at least two exons (multiexonic genes) and are potentially subjected to AS. In teosinte, 4,420 genes, which accounts for 32.52% of the studied multiexonic genes, were subjected to AS. A total of 19,059 AS events were identified in all teosinte libraries. After removing the same AS events in different libraries, 11,492 non-redundant AS events were detected in teosinte libraries, with an average of 2.26 AS events per gene. In maize, a total of 57,973 AS events, including 22,574 non-redundant AS events, were identified from 5,479 genes (45.5% of multiexonic genes) in all 10 maize libraries, with an average of 4.12 AS events per gene. Among different samples in the same species, the number of AS events and AS genes varied widely. In maize, 3,183 to 9,351 AS events involving 2,218 to 4,224 genes were identified. In teosinte, 2,514 to 4,088 AS events involving 1,822 to 2,660 genes were identified (Table 1).

**Table 1:** **Number of alternative splicing (AS) events and genes detected in maize and teosinte libraries**

	**AS-event**	**AS gene**	**AS events per gene**
B73	7,157	3,643	1.96
MO17	3,183	2,218	1.44
HZ4	4,810	2,927	1.64
YE478	5,656	3,320	1.70
ZI330	3,732	2,463	1.52
CML114	9,351	4,224	2.21
CML20	4,604	2,910	1.58
CML285	8,762	4,056	2.16
CML470	4,356	2,829	1.54
CML497	6,342	3,328	1.91
Maize-unique	32,058	7,775	4.12
K67-4	3,334	2,205	1.51
K69-3	2,545	1,844	1.38
WST92-1	4,088	2,660	1.54
K67-20	2,514	1,822	1.38
WS92-12	3,177	2,214	1.43
WS92-16	3,401	2,361	1.44
Teosinte-unique	12,386	5,479	2.26

Splicing Junction sites (SJs) were identified using Tophat software [26]. A total of 94,476 and 75,824 unique SJs were detected in all maize and teosinte samples. In maize, SJs were composed of 90,860 (96.17%) GT-AG, 2,638 (2.79%) GC-AG, and 978 (0.51%) AT-AC. Similarly, in teosinte, SJs were composed of 73,415 (96.82%) GT-AG, 1,822 (2.40%) GC-AG, and 587 (0.78%) AT-AC. The number of SJs ranged from 60,627 to 77,126 in maize libraries, and from 55,426 to 63,714 SJs in teosinte libraries (Additional file 1: Table S4). In both maize and teosinte, the canonical GT-AG pair represented the highest proportion of all splicing sites, followed by the GC-AG pair. This result was consistent with previous studies in other species [4,5,9].

To identify differences in splice site pairs between maize and teosinte, we compared unique splicing sites identified in the two species. A total of 97,146 SJs were identified. Of these, 73,154 (75.3%) were shared by the two species; 21,322 (21.9%) and 2,670 (2.7%) were specifically detected in maize and teosinte, respectively (Additional file 3: Figure S2). We also found that an average of 7.96 and 6.56 SJs in maize and teosinte, respectively, were detected for each gene. More SJs were detected in maize genes, likely because more AS transcripts were generated from maize genes relative to their orthologs in teosinte. This finding may also indicate that maize domestication is accompanied by an increase in the number of SJs rather than a change in splice site pairs.

Additionally, the four main AS types—IR, ES, AA, and AD—were analyzed. We found that IR accounted for 31.48% of all AS events (57,973) and was the most abundant AS type in maize, followed by AA (30.75%), AD (14.34%), and ES (11.49%). However, in the six teosinte libraries, AA accounted for 32.16% of all AS events (19,059) and was the most common AS type, followed by IR (28.46%), AD (13.65%), and ES (13.02%) (Table 2). These results demonstrate that both IR and AA could contribute to more than 60% of AS events and are consistent with findings in other plant species [4,5]. Notably, IR was the most common AS type in maize but not in teosinte.

**Table 2:** **Differences in alternative splicing (AS) events**

**AS type**	**Structure**	**Maize events**	**Maize ratio (%)**	**Teosinte events**	**Teosinte ratio (%)**
IR		18,242	31.48	5,424	28.46
ES		6,661	11.49	2,482	13.02
AD		8,310	14.34	2,602	13.65
AA		17,819	30.75	6,129	32.16
IR1 or IR2		1,149	1.98	558	2.93
IR1 + IR2		852	1.47	148	0.78
ES1 + ES2		628	1.08	249	1.31
Other		4,292	7.41	1,467	7.70

IR: intron retention; ES: exon skipping; AD: alternative donor site; AA: alternative acceptor site.

The effect of genomic features on AS were evaluated in both maize and teosinte. The result showed that exon number, gene length, totaling intron length and maximum intron length were highly positively correlated with the unique AS events, whereas GC content and exon lengths were negatively correlated with unique AS events (Table 3), indicating occurrence of AS may be strongly dependent on genic or genomic features. A similar result was reported in soybean [5]. Furthermore, the sequence length covered by each AS type was also analyzed in maize and teosinte. For IR, the retained intron length ranged from 52 to 5,099 bp in maize, but only 47 to 1,563 bp in teosinte. Although a few of the retained introns were longer in maize than teosinte, the frequency distribution of retained intron length was similar between the two species. Both species had retained introns that were substantially longer than 89 bp, which is longer than those in soybean [5]. For AA, the AS sequence length ranged from 2 to 3,591 bp in maize, but 2 to 1,283 bp in teosinte. Both maize and teosinte shared the most frequent AA length (4 bp). This result was consistent with previous findings in other species [5,27]. The most frequent skipped exon (ES) length was 72 bp in maize and 66 bp in teosinte. Moreover, the peaks in the distribution of sequence length for AD were 2 bp in maize and 5 bp in teosinte (Additional file 4: Figure S3). In general, the sequence length covered by each AS type did not significantly differ between maize and teosinte.

**Table 3:** **Relationship between genomic feature and unique AS events per gene in maize and teosinte**

	**Maize**		**Teosinte**	
**Features**	**Correlation coefficient**	**P-value**	**Correlation coefficient**	**P-value**
Exon number	0.335	1.14E-202	0.182	5.86E-42
Gene length (bp)	0.186	1.59E-61	0.091	1.18E-11
Totaling intron length (bp)	0.161	1.89E-45	0.086	2.69E-10
Maximum intron length (bp)	0.092	1.10E-15	0.060	9.08E-6
GC content (%)	−0.168	3.86E-50	−0.074	4.84E-8
Minimum exon length (bp)	−0.105	1.53E-20	−0.048	3.96E-04
Average exon length (bp)	−0.084	9.36E-14	−0.066	9.54E-07
Maximum exon length (bp)	−0.058	2.63E-07	−0.048	3.96E-04
Minimum intron length (bp)	−0.059	2.67E-7	−0.019	0.155
Average intron length (bp)	−0.013	0.25	−0.004	0.748

### Transposable elements and intron retention

To study the relationship between intron retention and transposable element (TE) insertion, 9,299 and 3,547 of the retained introns were retrieved from IR isoforms of maize and teosinte, and then searched against a repetitive DNA element database. However, 98 (1.05%) of the retained introns from 94 maize genes contained TE sequences that included *Copia* and *Gypsy* (Class I) as well as *Mutator* and *Stowaway* (Class II) (Table 4). Similarly, only nine (0.25%) of the retained introns from teosinte unigenes contained TE sequences, which included two *LINEs* (Class I), as well as *helitrons*, one *En-spm*, and one unknown TE (Class II). To validate that these AS transcripts rose by TE insertion, RNA sequencing data from kernels (15 d after pollination, DAP15) (SRP026161 www.ncbi.nlm.nih.gov/sra/) of 54 maize inbred lines were used to cross-validate these AS transcripts with TEs. Consequently, 57 of the 108 TEs inserted in the retained introns were repeatedly detected in the developing kernel (Additional file 5: Table S5), indicating that these AS events occurred in seedlings as well as developing kernels. The low frequency of TE sequences that harbor retained introns indicated that TE insertion might not be a critical cause of the high frequency of IR events in maize and teosinte. Alternatively, diverse TE sequences were detected in 98 and nine of the retained introns from maize and teosinte isoforms, respectively. Thus, some genes might be subjected to TE insertion, which potentially facilitates the origin of AS during maize domestication and improvement. TE insertion in introns may therefore be one of the most important mechanisms of intron retention.

**Table 4:** **Number and type of transposable elements (TEs) in retained introns**

**Class of TE**	**Type of TE**	**Number of TEs inserted in maize retained introns**	**Number of TEs inserted in teosinte retained introns**
I	LTR/*Copia*	11	0
I	LTR/*Gypsy*	11	0
I	LTR/*unknow*	2	0
I	LINE	30	2
II	*hAT*	14	0
II	*Helitron*	19	5
II	*En-spm*	5	1
II	*Stowaway*	5	0
II	Others	6	0
Total TEs		108	9
Number of the retained intron with TE		98	9
Total number of retained introns		9,299	3,547

### Difference in AS levels between maize and teosinte

To compare AS levels in maize and teosinte, we identified 2,766 genes that underwent AS in maize but not in teosinte. AS levels of 556 genes were four-fold higher in maize than in teosinte, and all 3,322 genes in maize had increased AS levels (Additional file 6: Table S6). However, only 542 genes had decreased AS levels in maize compared with teosinte. A total of 470 of these genes lost AS isoforms in maize, and the AS levels of 72 genes were four-fold lower in maize than in teosinte (Additional file 7: Table S7). The number of AS level-increased genes were 6.2-fold higher than the number of AS level-decreased genes in maize. This finding indicates that more genes have increased the complexity of AS but fewer genes have lost the complexity of AS during domestication from teosinte to maize.

**Figure 4:**
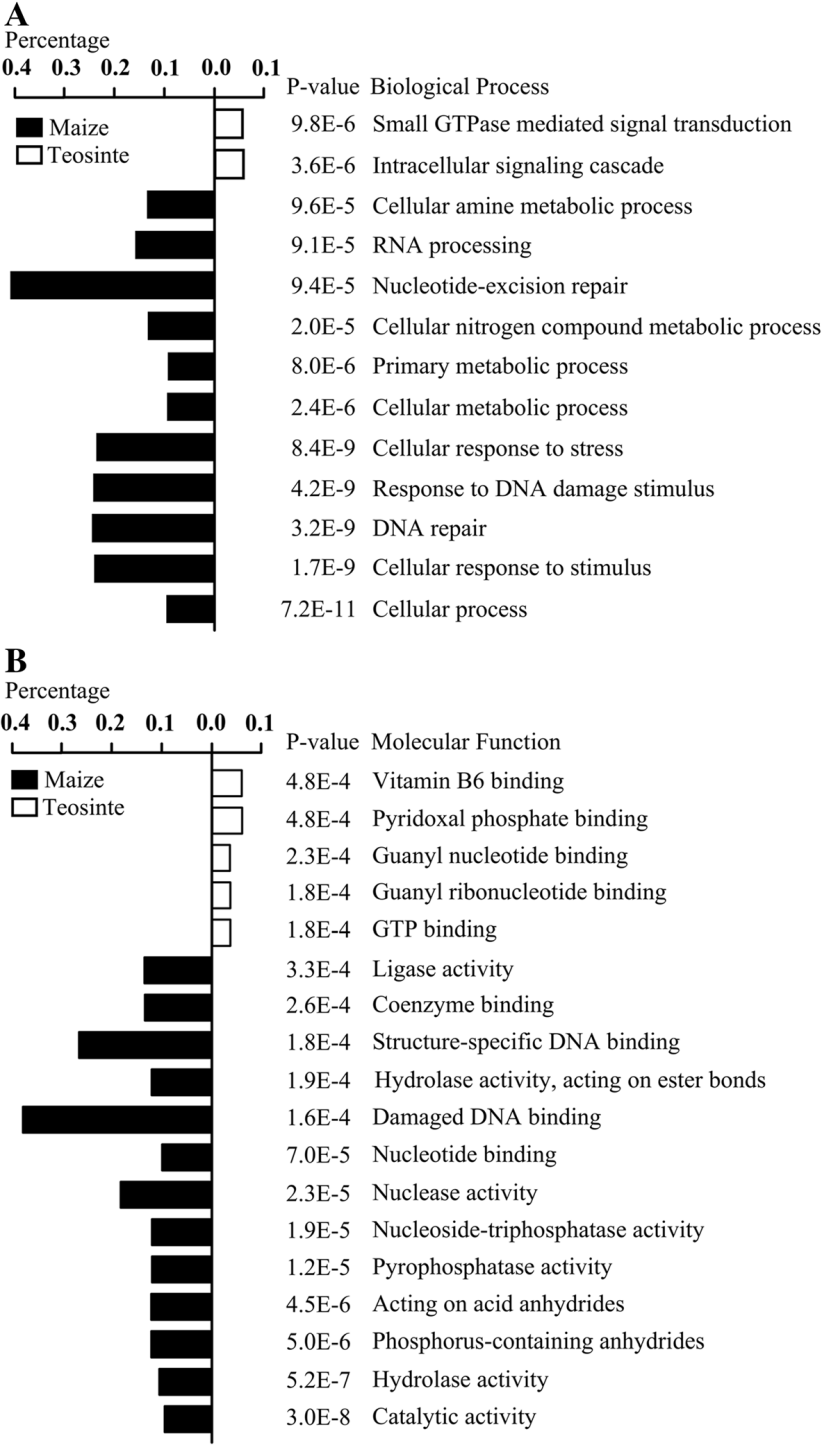
Gene ontology (GO) enrichment of all alternative splicing level-altered genes. GO enrichment was performed using agriGO. **(A)** Biological Process, **(B)** Molecular Function. The percentage is the ratio of enriched alternative splicing-altered genes to all genes in a given GO term using maize reference genes as background. The P-value denotes the enriched levels in a GO term, which were calculated using Fisher’s exact test.

To gain further insight into the role that these AS level-altered gene play, we performed a gene ontology (GO) analysis on the AS level-altered genes. We found that these AS level-increased genes were enriched in few biological processes: cellular response to stimuli, response to stress, and DNA damage stimuli (P-value ≤ 1E-8) and nucleotide excision repair (P-value = 9.4E-5). AS level-decreased genes were only enriched in intracellular signaling cascades and small GTPase-mediated signal transductions (P-value ≤ 1E-5) (Figure 4A). AS level-increased genes were also enriched in 13 molecular function terms, whereas AS level-decreased genes were enriched in only five molecular function terms (Figure 4B). Interestingly, AS level-decreased genes were enriched in vitamin B6 binding (P-value = 0.0005) and pyridoxal phosphate binding (P-value = 0.0005). Vitamin B6 is known to be involved in several biological processes, including amino acid metabolism; metabolism of fats and carbohydrates; and the ability to increase biotic and abiotic stresses, photosynthesis, and response to pathogens [28].

AS level-altered genes were mapped to genomic regions experiencing selection during maize domestication and improvement detected by Hufford et al. [22]. A total of 138 AS level-increased genes (Additional file 8: Table S8) and 15 AS level-decreased genes (Additional file 9: Table S9) were located in genomic regions with strong signals of selection. Perfectly matched potential candidates that were selected during maize improvement were inferred as putatively improved genes. Of these 138 genes, 108 did not show AS in teosinte but generated AS isoforms in maize, and 30 genes showed an average of a 7.36-fold increase in AS levels in maize relative to teosinte. In AS level-decreased genes, 11 genes lost different isoforms in maize, and four genes showed an average of 5.28-fold higher AS level in teosinte than in maize. These putatively improved genes included some transcription factors, such as NAC, Zinc finger, WRKY, and bZIP transcription factor genes, as well as some stress responsive genes, such as heat shock and drought-induced protein encoding genes. This indicates that these putatively improved genes may be involved in transcription regulation and stress responses.

## Discussion

Since Berget et al. [29] discovered intervening sequences, increasing evidence has revealed that AS plays an important role in transcription regulation and the origin of the functional diversity of eukaryotic genomes. AS can increase genome complexity without increasing genome content; thus, increasing AS levels should be positively correlated with organismal complexity. Using high throughput RNA sequencing, we comprehensively explored the number of isoforms and AS events, AS types, and SJs chosen in highly conserved subsets of genes in maize and teosinte. We found that the number of SJs was greater in maize than in teosinte, but the difference in the ratio of canonical and non-canonical SJs was not significantly different between the two species. More genes were subjected to AS in maize than in teosinte (7,775 vs. 5,479). Importantly, AS levels of orthologous genes greatly diverged between the two species and were higher in maize than in teosinte. More than 3,300 genes increased their AS levels but only 542 genes decreased AS levels, indicating that AS complexity is increased in modern maize relative to its progenitor. Nevertheless, two transcriptome assembly methods were used in the study, due to the ack of genomic information of teosinte, the difference of assembly methods potentially contributed to the AS level difference between maize and tesointe. A more comprehensive understanding of teosinte AS landscape is still dependent on the reference-based transcriptome assembly.

A large-scale study using 39 million expressed sequence tags from 47 eukaryotic species revealed that the proportion of AS genes and the average number of AS isoforms per gene (AS level) have gradually increased over the past 1.4 billion years; thus, AS complexity can be considered a strong predictor of organismal complexity [30]. A similar phenomenon is also observed in vertebrate species [31], with all of these results indicating that there is a general trend of AS increasing levels in eukaryotic evolution.

During crop domestication and improvement, hundreds of genes had altered expression levels and co-expression relationships [32,33], and dozens of differentially expressed genes show significant enrichment for targets of selection, which indicates that expression level selection of specific sets of genes is an important mechanism in maize evolution. In this study, we found that 138 AS level-increased genes were located in regions that were targets of selection during maize improvement and putatively inferred to be improved genes. However, none were located in potential domestication-related genes detected by genome sequencing [22]. This result demonstrates that the increase in AS level might be a result of improving flexibility and the degree of regulation at the transcriptional and post-transcriptional levels during maize improvement. Thus, this AS level increase may be an important mechanism of maize evolution.

GO enrichment revealed that the 3,322 AS level-increased genes were enriched in a few biological processes and molecular functions (Figure 4). For example, GRMZM2G014653, GRMZM2G018436, GRMZM2G001887, GRMZM2G054277, GRMZM2G110116, and GRMZM2G113950 all encode a group of NAC-domain proteins that play important roles in the biotic and abiotic stress response regulation [34]. GRMZM2G022359, GRMZM2G171179, and GRMZM2G363052 encode ERF transcription factors that are involved in diverse abiotic stress responses and developmental process regulation [35]. GRMZM2G088064 encodes alanine aminotransferase, and both GRMZM2G094712 and GRMZM2G067265 encode aspartate aminotransferases; all are induced by stress factors that facilitate the acquisition of somatic embryogenesis capacities [36]. GRMZM2G002656, AC230011.2_FG002, and seven other genes encode proteins with an NB-ARC domain, which is a central nucleotide-binding domain of a resistance (R) protein that is involved in pathogen recognition and subsequent innate immune response activation [37]. GRMZM2G003635, GEMZM2G021687, GRMZM2G031637, and 29 other heat shock protein-encoding genes were also detected. All of these genes play potential roles in various biotic and abiotic stress responses. Moreover, GRMZM2G094712, GRMZM2G088064, GRMZM2G067265, and 11 other genes are involved in oxygenic photosynthesis; GRMZM2G382914, GRMZM2G003724, GRMZM2G089136, and nine other genes are involved in the Calvin–Benson–Bassham cycle. Some flowering-related genes also showed AS level increase in maize, such as GRMZM2G026223 (a MADS-box transcription factor), GRMZM2G402862 (a bZIP transcription factor), and GRMZM2G400167 (a FT-like protein). They were also reported to be involved in flowering regulation in maize (Additional file 10: Table S10). In particular, 151 putative improved genes were enriched in transcription regulation and stress response.

During domestication from teosinte to modern maize, natural and artificial selection occurred in two phases: domestication and improvement. In the domestication phase, selection focused on making maize cultivatable and improving seed access. Several regulatory genes, such as *tga1* [16], *tb1* [17], and *ra1* [20], are thought to be responsible for the major morphology changes from teosinte to landrace. In the improvement phase, selection focused on yield, grain quality, and agro-ecosystem adaptations [38]. We suggest that an increase in AS complexity as well as changes in the expression and co-expression profile are likely responsible for increased adaptation and organic matter accumulation by modern maize breeding.

## Conclusions

In this study, more than 13,000 orthologous genes were identified by comparative transcriptome analysis of maize and teosinte, and AS profiles of these orthologous genes were then identified. We found that both maize and teosinte shared similar AS mechanisms, but more genes have increased AS complexity during domestication from teosinte to maize. In particular, a subset of AS level-increased genes that mainly encode transcription factors and stress-responsive proteins may have been selected during maize improvement.

## Methods

### Plant materials

Seeds from ten maize inbred lines (including five temperate and five tropical lines) and six teosinte, including three *Zea mays* ssp. *parviglumis* and three *Zea mays* ssp. *mexicana* accessions (Additional file 1: Table S1), were separately germinated in an incubator with a 12-hour dark–light cycle. The germinated seeds were grown in a greenhouse for 2 weeks, and six seedlings, including shoots and roots for each line/accession, were harvested for RNA extraction using Trizol reagent (Invitrogen, Carlsbad, CA, USA).

### Library construction and transcriptome sequencing

Total RNA was denatured at 65°C and extracted twice with Sera-mag Magnetic Oligo(dT) Beads (Thermo Fisher Scientific. Wilmington, DE, USA). The purified mRNA was then treated with a divalent cation solution followed by ethanol precipitation. The re-suspended mRNAs were then used for first-strand cDNA synthesis using reverse transcription with random primers, followed by second-strand cDNA synthesis using DNA Polymerase I and RNase H. The double-stranded cDNA fragments were then end-repaired with T4 DNA polymerase, and an “A” base was added to the blunt cDNA fragments using Klenow DNA polymerase. The adenylated cDNA was purified with a MinElute PCR Purification Kit (QIAGEN, Valencia, CA, USA). Illumina’s paired-end oligo adapters were then added to the cDNA fragments with T4 ligase, followed by purification using a QIAquick PCR Purification Kit (QIAGEN). The library was eluted in 10 μL of Nuclease-free water followed by purification on 2% agarose gel. A 250 ± 25 bp gel slice was excised, and the cDNAs were eluted using a QIAquick Gel Extraction Kit (QIAGEN). The eluted cDNAs were then enriched by 18 PCR cycles followed by gel purification. The recovered cDNAs were quantified with a Nanodrop (Thermo Fisher Scientific) and a TBS-380 mini-fluorometer (Turner Biosystems, Sunnyvale, CA, USA) using Picogreen® dsDNA quantization reagent (Invitrogen). The concentration of the sample was adjusted to ~10 nM.

The cDNA library was sequenced on an Illumina Hiseq 2000 platform (San Diego, CA, USA). Typically, a paired-end sequencing run with an approximately 75–100-nt read length is performed. All of the sequencing was completed by LC Sciences (Houston, TX, USA). All sequencing data have been deposited in the NCBI Sequence Read Archive.

### Read processing and transcriptome assembly

Raw RNA sequencing data were processed using FASTX-Toolkit (http://hannonlab.cshl.edu/fastx_toolkit/). Low quality (Phred quality score Q < 20) nucleotides were trimmed from the 3′-end of the reads and were masked by “N.” Reads < 40 bp were also discarded.

Without a reference genome, cleaning reads from each teosinte accession were *de novo* assembled into contigs with Trans-ABySS [39], which uses a multiple K-mer strategy to assemble a transcriptome. This is considered the best method of *de novo* transcriptome assembly [40]. Contigs larger than 100 bp were used for further study. To obtain more information about the transcriptome of *Zea mays* ssp. *parviglumis* and *Zea mays* ssp. *mexicana*, we first pooled three transcriptomes from each of the subspecies, all redundant contigs were removed, and remaining contigs were further assembled into unigenes using CAP3 [41]. Sequences with 98% identity were used to control for sequence variation and genomic heterogeneity. The isoforms from each maize inbred line were reconstructed based on the B73 reference genome (ZmB73_RefGen_v2) [42].

### Variant calling and genetic diversity analysis

High quality reads from each library were first mapped to the B73 reference genome using Burrows Wheeler Aligner (BWA, version 0.6.1) [43], which allows a maximum of two mismatches. Bcftools [44] was used for single nucleotide polymorphism (SNP) and insertion/deletion(indel) calling. SNPs and indels with quality scores > 20 and depths > 15 were considered high quality variants. High quality SNPs were then used to reconstruct phylogenetic relationships using the neighbor-joining method with 1000 bootstrap replicates in the PHYLIP package (version 3.6.9) [45], and MEGA 6.0 was used to generate the NJ-tree image [46].

### Orthologous gene identification

The reciprocal best blast hit strategy (RBH) was used to identify orthologous gene pairs in maize and teosinte. The *Zea mays* ssp*. parviglumis* and *Zea mays* ssp. *mexicana* unigenes were separately blasted against the maize reference cDNAs (ZmB73_5b_FGS_cdna). Similarly, maize reference cDNAs were also aligned against *Zea mays* ssp*. parviglumis* and *Zea mays* ssp*. mexicana* unigenes. Custom perl scripts from Harvard University FAS Center for Systems Biology (http://sysbio.harvard.edu) were used to extract the reciprocal best hits with e-values ≤ 1E-10. Furthermore, the shared reciprocal best hits between *Zea mays* ssp. *parviglumis* and *Zea mays* ssp. *mexicana* were extracted to represent teosinte transcripts and were defined as orthologous genes of maize.

### AS profile analyses of maize and teosinte

Tophat was used for SJs detection with default parameters settings [26]. To reduce the false discovery rate, those SJs that supported more than 10 reads were retained for further analysis. To identify AS events that occur in orthologous genes in maize and teosinte, Cufflinks was used to reconstruct empirical transcripts [47]. The minimum isoform fraction was set to 0.05, the small anchor fraction of spliced reads was set to 0.01, and the minimum and maximum size of introns were set to 30 and 100,000 bp, respectively. The assembled isoforms were mapped to the corresponding B73 gene model using Cuffcompare, which is included in the Cufflinks software [47]. AS event identification was performed with ASTALAVISTA and classified into different types as described by Foissac and Sammeth [48].

To estimate the effect of sequencing depths on the power of alternative splicing detection, a simulation was performed to detect the AS genes and AS events using libraries with different sequencing depths (0.5–4.5 Gb) that were randomly sampled from the Ye478 library with 50 replicates. To compare the difference in alternative splicing between maize and teosinte, the AS level was defined as the average number of AS events per gene within a species. To prevent AS level bias that is caused by gene expression level, the differentially expressed genes in the two species were detected by Cuffdiff using the default parameters. Only genes with similar expression levels were used to compare the AS level differences in the two species [47]. If AS events were identified in one species but absent in another species or four-fold higher in one species than in another species, we determined that the AS level of the gene was markedly different between these two species. Furthermore, GO enrichment of these AS level-altered genes was performed using agriGO [49]. Moreover, the transposable elements that harbor retained introns were found by searching against the Genetic Information Research Institute’s Repetitive DNA element database (http://www.girinst.org/repbase/) using RepeatMasker (http://www.repeatmasker.org/).

### Availability

RNA-seq data from this publication have been submitted to the National Center for Biotechnology Information Sequence Read Archive database (SRP051572: SRX824561, SRX824579–824593). Phylogenetic data (SNPs in HapMap format, alignment file in phylip sequential format and neighbor-joining tree files) have been deposited in the Dryad Digital Repository (http://dx.doi.org/10.5061/dryad.tk2fn).

## Additional files

Additional file 1:
**Supplemental files.** This file contains Tables S1 to S4. **Table S1.** Sequenced material and read information. **Table S2.** Summary of *de novo* assembled teosinte unigenes. **Table S3.** Summary of maize transcriptomes assembled based on reference genome. **Table S4.** Splicing junction site number and types in maize and teosinte. Additional file 2: Figure S1. Single nucleotide polymorphism (SNP) density per gene and insertion/deletion (indel) length distribution. A) SNP density per gene. The X-axis denotes the number of SNPs per gene. The Y-axis denotes gene numbers. B) Length distribution of Indels. Additional file 3: Figure S2. The consistency of splicing junction sites in maize and teosinte. Additional file 4: Figure S3. Sequence length distribution of the difference types of AS events. A) Frequency distribution of alternative donor (AD) length; B) Frequency distribution of alternative acceptor (AA) length; C) Frequency distribution of retained intron (IR) length; and D) Frequency distribution of skipped exon (ES) length. Additional file 5: Table S5. Transposable elements (TEs) in retained introns and cross-validation. Additional file 6: Table S6. Annotation of alternative splicing level-increased genes in maize inbred lines. Additional file 7: Table S7. Annotation about alternative splicing level-decreased genes in maize inbred lines. Additional file 8: Table S8. Alternative splicing level-increased genes in maize with strong signals of selection from maize improvement. Additional file 9: Table S9. Alternative splicing level-decreased genes in maize with strong signals of selection from maize improvement. Additional file 10: Table S10. Alternative splicing level-increased genes in maize that are related to flowering.

## Abbreviations

AS: Alternative splicing
ES: Exon skipping
AD: Alternative donor site
AA: Alternative acceptor site
IR: Intron retention
TE: Transposable element
GO: Gene ontology
SNP: Single nucleotide polymorphism
indel: Insertion/deletion
bp: Base pair

## References

[1] Staiger D, Brown JW (2013). Alternative splicing at the intersection of biological timing, development, and stress responses. Plant Cell.

[2] Gilbert W (1978). Why genes in pieces?. Nature.

[3] Wang ET, Sandberg R, Luo S, Khrebtukova I, Zhang L, Mayr C (2008). Alternative isoform regulation in human tissue transcriptomes. Nature.

[4] Marquez Y, Brown JW, Simpson C, Barta A, Kalyna M (2012). Transcriptome survey reveals increased complexity of the alternative splicing landscape in Arabidopsis. Genome Res.

[5] Shen Y, Zhou Z, Wang Z, Li W, Fang C, Wu M (2014). Global dissection of alternative splicing in paleopolyploid soybean. Plant Cell.

[6] Lu X, Chen D, Shu D, Zhang Z, Wang W, Klukas C (2013). The differential transcription network between embryo and endosperm in the early developing maize seed. Plant Physiol.

[7] Keren H, Lev-Maor G, Ast G (2010). Alternative splicing and evolution: diversification, exon definition and function. Nat Rev Genet.

[8] Walters B, Lum G, Sablok G, Min XJ (2013). Genome-wide landscape of alternative splicing events in Brachypodium distachyon. DNA Res.

[9] Li Q, Xiao G, Zhu YX (2014). Single-nucleotide resolution mapping of the Gossypium raimondii transcriptome reveals a new mechanism for alternative splicing of introns. Mol Plant.

[10] Kornblihtt AR, Schor IE, Allo M, Dujardin G, Petrillo E, Munoz MJ (2013). Alternative splicing: a pivotal step between eukaryotic transcription and translation. Nat Rev Mol Cell Bio.

[11] Balasubramanian S, Sureshkumar S, Lempe J, Weigel D (2006). Potent induction of Arabidopsis thaliana flowering by elevated growth temperature. PLoS Genet.

[12] Filichkin SA, Priest HD, Givan SA, Shen R, Bryant DW, Fox SE (2010). Genome-wide mapping of alternative splicing in Arabidopsis thaliana. Genome Res.

[13] Lin Z, Li X, Shannon LM, Yeh CT, Wang ML, Bai G (2012). Parallel domestication of the Shattering1 genes in cereals. Nat Genet.

[14] Matsuoka Y, Vigouroux Y, Goodman MM, Sanchez GJ, Buckler E, Doebley J (2002). A single domestication for maize shown by multilocus microsatellite genotyping. Proc Natl Acid Sci USA.

[15] Gaut BS, Le Thierry d’Ennequin M, Peek AS, Sawkins MC (2000). Maize as a model for the evolution of plant nuclear genomes. Proc Natl Acid Sci USA.

[16] Wang H, Nussbaum-Wagler T, Li B, Zhao Q, Vigouroux Y, Faller M (2005). The origin of the naked grains of maize. Nature.

[17] Doebley J, Stec A, Hubbard L (1997). The evolution of apical dominance in maize. Nature.

[18] Gallavotti A, Zhao Q, Kyozuka J, Meeley RB, Ritter MK, Doebley JF (2004). The role of barren stalk1 in the architecture of maize. Nature.

[19] Vollbrecht E, Springer PS, Goh L, Buckler ES, Martienssen R (2005). Architecture of floral branch systems in maize and related grasses. Nature.

[20] Bomblies K, Doebley JF (2006). Pleiotropic effects of the duplicate maize FLORICAULA/LEAFY genes *zfl1* and *zfl2* on traits under selection during maize domestication. Genetics.

[21] Yamasaki M, Tenaillon MI, Bi IV, Schroeder SG, Sanchez-Villeda H, Doebley JF (2005). A large-scale screen for artificial selection in maize identifies candidate agronomic loci for domestication and crop improvement. Plant Cell.

[22] Hufford MB, Xu X, van Heerwaarden J, Pyhajarvi T, Chia JM, Cartwright RA (2012). Comparative population genomics of maize domestication and improvement. Nat Genet.

[23] Swanson-Wagner R, Briskine R, Schaefer R, Hufford MB, Ross-Ibarra J, Myers CL (2012). Reshaping of the maize transcriptome by domestication. Proc Natl Acid Sci USA.

[24] Lemmon ZH, Bukowski R, Sun Q, Doebley JF (2014). The role of cis regulatory evolution in maize domestication. PLoS Genet.

[25] Liu R, Loraine AE, Dickerson JA (2014). Comparisons of computational methods for differential alternative splicing detection using RNA-seq in plant systems. BMC Bioinformatics.

[26] Trapnell C, Pachter L, Salzberg SL (2009). TopHat: discovering splice junctions with RNA-Seq. Bioinformatics.

[27] Campbell MA, Haas BJ, Hamilton JP, Mount SM, Buell CR (2006). Comprehensive analysis of alternative splicing in rice and comparative analyses with Arabidopsis. BMC Genomics.

[28] Mooney S, Hellmann H (2010). Vitamin B6: killing two birds with one stone?. Phytochemistry.

[29] Berget SM, Moore C, Sharp PA (1977). Spliced segments at the 5′terminus of adenovirus 2 late mRNA. Proc Natl Acid Sci USA.

[30] Chen L, Bush SJ, Tovar-Corona JM, Castillo-Morales A, Urrutia AO (2014). Correcting for differential transcript coverage reveals a strong relationship between alternative splicing and organism complexity. Mol Biol Evol.

[31] Barbosa-Morais NL, Irimia M, Pan Q, Xiong HY, Gueroussov S, Lee LJ (2012). The evolutionary landscape of alternative splicing in vertebrate species. Science.

[32] Meyer RS, DuVal AE, Jensen HR (2012). Patterns and processes in crop domestication: an historical review and quantitative analysis of 203 global food crops. New Phytol.

[33] Koenig D, Jimenez-Gomez JM, Kimura S, Fulop D, Chitwood DH, Headland LR (2013). Comparative transcriptomics reveals patterns of selection in domesticated and wild tomato. Proc Natl Acid Sci USA.

[34] Nuruzzaman M, Sharoni AM, Kikuchi S (2013). Roles of NAC transcription factors in the regulation of biotic and abiotic stress responses in plants. Front Microbiol.

[35] Piyatrakul P, Putranto R-A, Martin F, Rio M, Dessailly F, Leclercq J (2012). Some ethylene biosynthesis and AP2/ERF genes reveal a specific pattern of expression during somatic embryogenesis in Hevea brasiliensis. BMC Plant Biol.

[36] Karami O, Saidi A (2010). The molecular basis for stress-induced acquisition of somatic embryogenesis. Mol Biol Rep.

[37] van Ooijen G, Mayr G, Kasiem MM, Albrecht M, Cornelissen BJ, Takken FL (2008). Structure-function analysis of the NB-ARC domain of plant disease resistance proteins. J Exp Bot.

[38] Buckler ES, Gaut BS, McMullen MD (2006). Molecular and functional diversity of maize. Curr Opin Plant Biol.

[39] Robertson G, Schein J, Chiu R, Corbett R, Field M, Jackman SD (2010). De novo assembly and analysis of RNA-seq data. Nat Methods.

[40] Zhao QY, Wang Y, Kong YM, Luo D, Li X, Hao P (2011). Optimizing de novo transcriptome assembly from short-read RNA-Seq data: a comparative study. BMC Bioinformatics.

[41] Huang X, Madan A (1999). CAP3: A DNA sequence assembly program. Genome Res.

[42] Schnable PS, Ware D, Fulton RS, Stein JC, Wei F, Pasternak S (2009). The B73 maize genome: complexity, diversity, and dynamics. Science.

[43] Li H, Durbin R (2009). Fast and accurate short read alignment with burrows-wheeler transform. Bioinformatics.

[44] Li H, Handsaker B, Wysoker A, Fennell T, Ruan J, Homer N, Marth G (2009). The sequence alignment/Map format and SAM tools. Bioinformatics.

[45] Felsenstein J (1989). PHYLIP-phylogeny inference package (version 3.2). Cladistics.

[46] Tamura K, Stecher G, Peterson D, Filipski A, Kumar S (2013). MEGA6: molecular evolutionary genetics analysis version 6.0. Mol Biol Evol.

[47] Trapnell C, Williams BA, Pertea G, Mortazavi A, Kwan G, van Baren MJ (2010). Transcript assembly and quantification by RNA-Seq reveals unannotated transcripts and isoform switching during cell differentiation. Nat Biotechnol.

[48] Foissac S, Sammeth M (2007). ASTALAVISTA: dynamic and flexible analysis of alternative splicing events in custom gene datasets. Nucleic Acids Res.

[49] Du Z, Zhou X, Ling Y, Zhang Z, Su Z (2010). agriGO: a GO analysis toolkit for the agricultural community. Nucleic Acids Res.

