# Supplementary material for: Comparative transcriptomics uncovers alternative splicing changes and signatures of selection from maize improvement
Source: BMC Genomics. 2015 May 8;16(1):363. doi: 10.1186/s12864-015-1582-5 (PMC4433066; doi:10.1186/s12864-015-1582-5)
Supplement: Additional file 1: — Supplemental files. This file contains Tables S1 to S4. Table S1. Sequenced material and read information. Table S2. Summary of de novo assembled teosinte unigenes. Table S3. Summary of maize transcriptomes assembled based on reference genome. Table S4. Splicing junction site number and types in maize and teosinte. [file 12864_2015_1582_MOESM1_ESM.docx]

**Table S1 Sequenced material and read information**

| Species | Name | Sources | Raw data sets  (G bp) |
| --- | --- | --- | --- |
| *Zea mays* ssp. *mexicana* | K67-4 | Mexico, Mexico | 2.66 |
| *Zea mays* ssp. *mexicana* | K69-3 | Michoacan, Mexico | 2.71 |
| *Zea mays* ssp. *mexicana* | WST92-1 | Durango, Mexico | 3.25 |
| *Zea mays* ssp. *parviglumis* | K67-20 | Michoacan, Mexico | 2.71 |
| *Zea mays* ssp. *parviglumis* | WS92-12 | Guerrero, Mexico | 2.69 |
| *Zea mays* ssp. *parviglumis* | WS92-16 | Michoacan, Mexico | 3.08 |
| *Zea mays* ssp. *mays* | B73 | Iowa, USA | 2.22 |
| *Zea mays* ssp. *mays* | MO17 | Missouri, USA | 3.42 |
| *Zea mays* ssp. *mays* | HZ4 | China | 5.28 |
| *Zea mays* ssp. *mays* | ZI330 | China | 3.60 |
| *Zea mays* ssp. *mays* | YE478 | China | 5.23 |
| *Zea mays* ssp. *mays* | CML20 | CIMMYT, Mexico | 5.81 |
| *Zea mays* ssp. *mays* | CML114 | CIMMYT, Mexico | 5.64 |
| *Zea mays* ssp. *mays* | CML285 | CIMMYT, Mexico | 4.61 |
| *Zea mays* ssp. *mays* | CML470 | CIMMYT, Mexico | 3.14 |
| *Zea mays* ssp. *mays* | CML497 | CIMMYT, Mexico | 2.30 |

**Table S2 Summary of *de novo* assembled teosinte unigenes**

| Material | Contig number | Long transcripts number(>=1000bp) | Maximum length(bp) | N50  (bp) | N90  (bp) |
| --- | --- | --- | --- | --- | --- |
| K67-4 | 55069 | 8577 | 8770 | 937 | 217 |
| K69-3 | 69069 | 7425 | 7284 | 731 | 185 |
| WST92-1 | 95668 | 10899 | 8342 | 802 | 176 |
| K67-20 | 88243 | 4954 | 7384 | 493 | 151 |
| WS92-12 | 72255 | 8912 | 7690 | 809 | 185 |
| WS92-16 | 88640 | 8050 | 7829 | 663 | 165 |
| *Mexicana* | 118886 | 17532 | 8901 | 983 | 193 |
| *Parviglumis* | 123759 | 14488 | 8296 | 834 | 172 |

**Table S3 Summary of maize transcriptomes assembled based on reference genome**

| Line | Isoform number | Long transcripts number (>1000bp) | Maximum length (bp) | N50  (bp) | N90  (bp) |
| --- | --- | --- | --- | --- | --- |
| B73 | 39441 | 25811 | 16052 | 1890 | 843 |
| MO17 | 49677 | 17454 | 13113 | 1314 | 467 |
| HZ4 | 52827 | 24445 | 13242 | 1656 | 600 |
| ZI330 | 47210 | 18983 | 13425 | 1457 | 528 |
| YE478 | 52763 | 25608 | 14246 | 1687 | 633 |
| CML20 | 53105 | 21417 | 13206 | 1528 | 523 |
| CML114 | 50073 | 27304 | 13934 | 1805 | 713 |
| CML285 | 47937 | 25405 | 11912 | 1738 | 692 |
| CML470 | 47682 | 20243 | 13505 | 1538 | 555 |
| CML497 | 39630 | 21672 | 13284 | 1748 | 715 |

**Table S4 Splicing junction site number and types in maize and teosinte**

|  | GT-AG | GC-AG | AT-AC | Total | AS number | Average |
| --- | --- | --- | --- | --- | --- | --- |
| B73 | 65284 | 1144 | 177 | 66605 | 10898 | 6.11 |
| MO17 | 59214 | 1171 | 242 | 60627 | 10974 | 5.52 |
| HZ4 | 71713 | 1469 | 380 | 73562 | 11401 | 6.45 |
| YE478 | 71162 | 1473 | 348 | 72983 | 11269 | 6.48 |
| ZI330 | 61467 | 1166 | 243 | 62876 | 10804 | 5.82 |
| CML114 | 75247 | 1536 | 343 | 77126 | 11122 | 6.93 |
| CML20 | 66942 | 1374 | 352 | 68668 | 11344 | 6.05 |
| CML285 | 70690 | 1400 | 304 | 72376 | 11141 | 6.50 |
| CML470 | 64195 | 1252 | 248 | 65695 | 10936 | 6.01 |
| CML497 | 64664 | 1137 | 196 | 65997 | 10844 | 6.09 |
| Maize-unique | 90860 | 2638 | 978 | 94476 | 11866 | 7.96 |
| K67-4 | 57892 | 1126 | 248 | 59266 | 10784 | 5.50 |
| K69-3 | 54180 | 1021 | 225 | 55426 | 10611 | 5.22 |
| WST92-1 | 62209 | 1229 | 276 | 63714 | 11010 | 5.79 |
| K67-20 | 54143 | 1100 | 224 | 55464 | 10627 | 5.22 |
| WS92-12 | 58082 | 1135 | 216 | 59433 | 10849 | 5.48 |
| WS92-16 | 57557 | 1171 | 247 | 58975 | 10849 | 5.44 |
| Teosinte-unique | 73415 | 1822 | 587 | 75824 | 11566 | 6.56 |
