# Supplementary figures and images for: Comparative transcriptomics uncovers alternative splicing changes and signatures of selection from maize improvement
Source: BMC Genomics. 2015 May 8;16(1):363. doi: 10.1186/s12864-015-1582-5 (PMC4433066; doi:10.1186/s12864-015-1582-5)

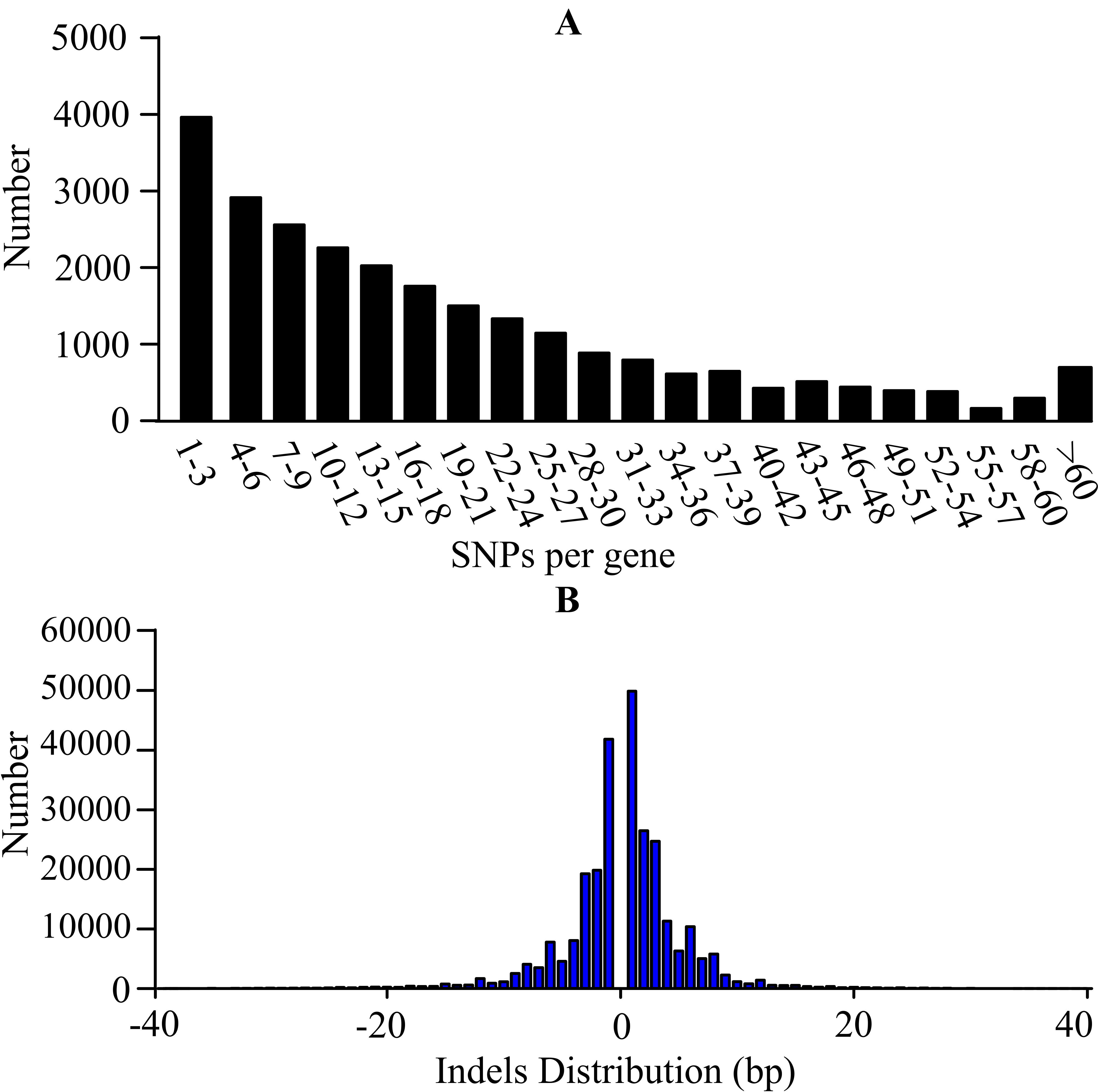

Supplement: Additional file 2: Figure S1. — Single nucleotide polymorphism (SNP) density per gene and insertion/deletion (indel) length distribution. A) SNP density per gene. The X-axis denotes the number of SNPs per gene. The Y-axis denotes gene numbers. B) Length distribution of Indels. [file 12864_2015_1582_MOESM2_ESM.tiff]

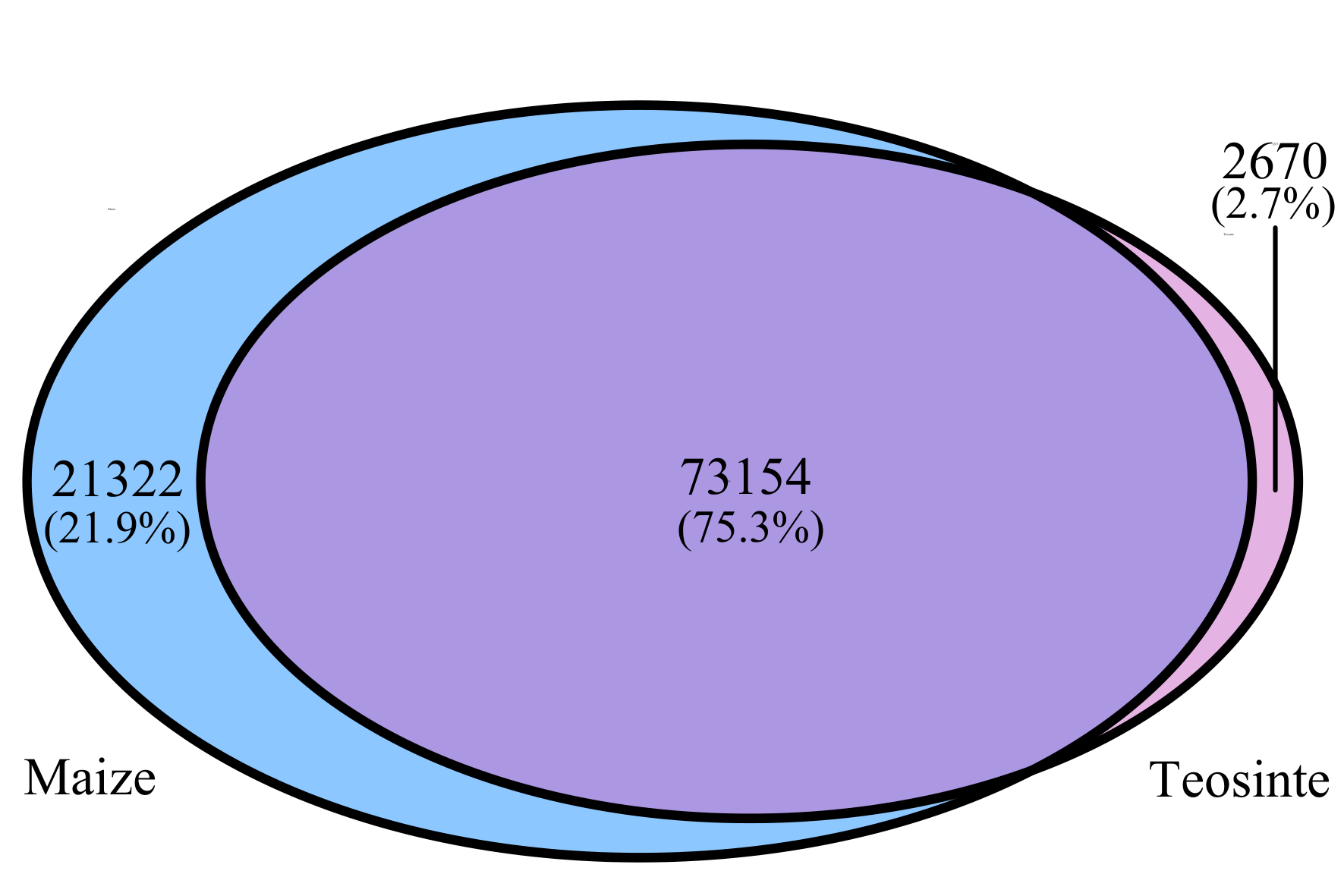

Supplement: Additional file 3: Figure S2. — The consistency of splicing junction sites in maize and teosinte. [file 12864_2015_1582_MOESM3_ESM.tiff]

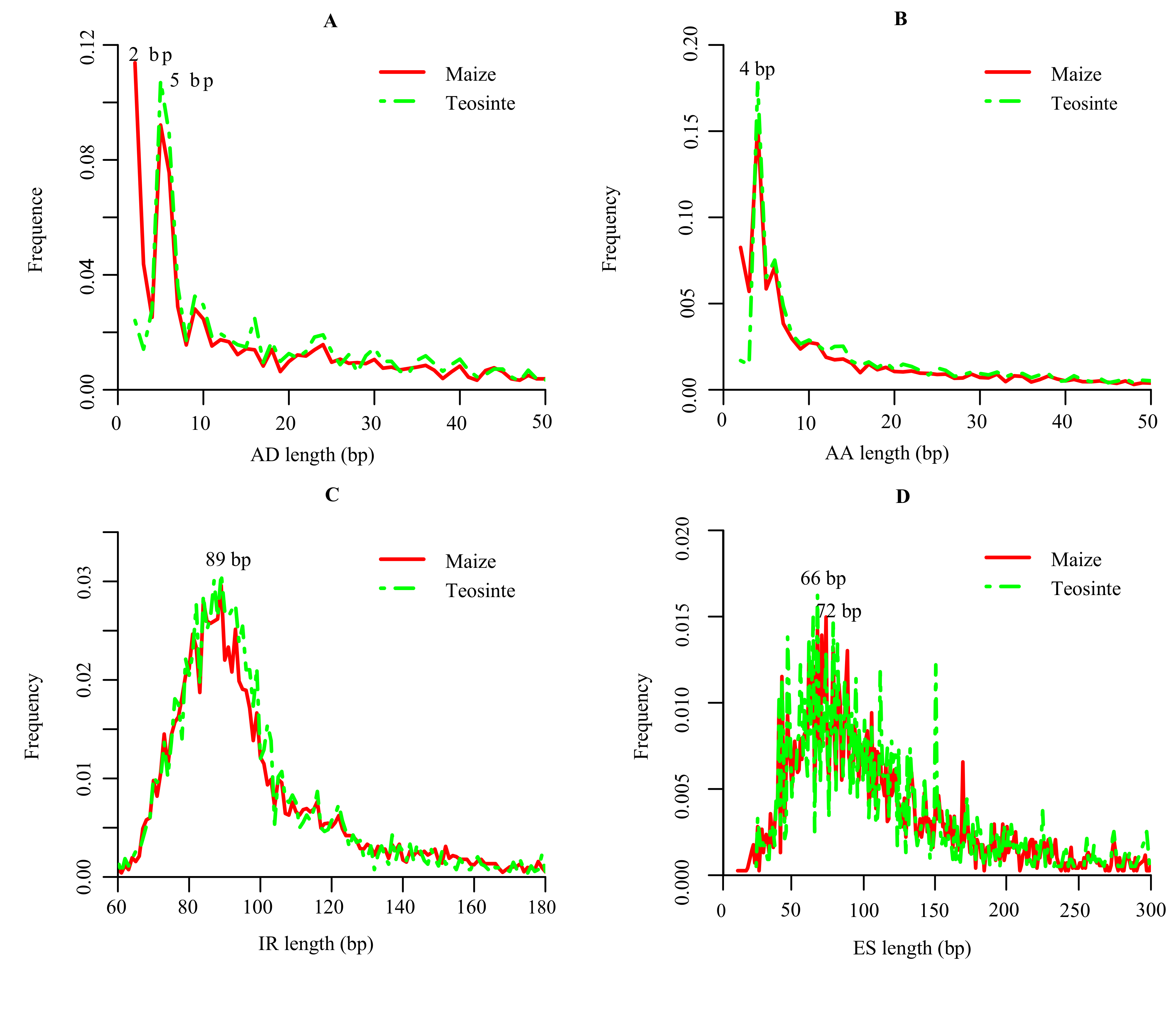

Supplement: Additional file 4: Figure S3. — Sequence length distribution of the difference types of AS events. A) Frequency distribution of alternative donor (AD) length; B) Frequency distribution of alternative acceptor (AA) length; C) Frequency distribution of retained intron (IR) length; and D) Frequency distribution of skipped exon (ES) length. [file 12864_2015_1582_MOESM4_ESM.tiff]
